# Quantification of H_2_^17^O by ^1^H-MR imaging at 3 T: a feasibility study

**DOI:** 10.1186/s41747-021-00246-w

**Published:** 2021-12-29

**Authors:** Luis Martí-Bonmatí, Alejandro Rodríguez-Ortega, Amadeo Ten-Esteve, Ángel Alberich-Bayarri, Bernardo Celda, Eduardo Ferrer

**Affiliations:** 1 Biomedical Imaging Research Group (GIBI230) at La Fe Health Research Institute and Imaging La Fe node at Distributed Network for Biomedical Imaging (ReDIB) Unique Scientific and Technical Infrastructures (ICTS), La Fe University and Polytechnic Hospital, Av. Fernando Abril Martorell, 106, Torre E, Planta 0, 46026 Valencia, Spain; 2 Quantitative Imaging Biomarkers in Medicine, QUIBIM SL, Valencia, Spain; 3 Physical Chemistry Department, University of Valencia, Valencia, Spain; 4 Radiotherapy Department, Hospital Clínico Universitario, Valencia, Spain

**Keywords:** Brain, Magnetic resonance imaging, Oxygen-17, Phantoms (imaging), Rats

## Abstract

**Background:**

Indirect ^1^H-magnetic resonance (MR) imaging of ^17^O-labelled water allows imaging *in vivo *dynamic changes in water compartmentalisation. Our aim was to describe the feasibility of indirect ^1^H-MR methods to evaluate the effect of H_2_^17^O on the MR relaxation rates by using conventional a 3-T equipment and voxel-wise relaxation rates.

**Methods:**

MR images were used to calculate the R1, R2, and R2* relaxation rates in phantoms (19 vials with different H_2_^17^O concentrations, ranging from 0.039 to 5.5%). Afterwards, an experimental animal pilot study (8 rats) was designed to evaluate the *in vivo* relative R2 brain dynamic changes related to the intravenous administration of ^17^O-labelled water in rats.

**Results:**

There were no significant changes on the R1 and R2* values from phantoms. The R2 obtained with the turbo spin-echo T2-weighted sequence with 20-ms echo time interval had the higher statistical difference (0.67 s^−1^, interquartile range 0.34, *p* < 0.001) and Spearman correlation (rho 0.79). The R2 increase was adjusted to a linear fit between 0.25 and 5.5%, represented with equation R2 = 0.405 concentration + 0.3215. The highest significant differences were obtained for the higher concentrations (3.1–5.5%). The rat brain MR experiment showed a mean 10% change in the R2 value after the H_2_^17^O injection with progressive normalisation.

**Conclusions:**

Indirect ^1^H-MR imaging method is able to measure H_2_^17^O concentration by using R2 values and conventional 3-T MR equipment. Normalised R2 relative dynamic changes after the intravenous injection of a H_2_^17^O saline solution provide a unique opportunity to map water pathophysiology *in vivo*, opening the analysis of aquaporins status and modifications by disease at clinically available 3-T proton MR scanners.

## Key points

- H_2_^17^O concentrations modify R2 values in conventional standard-of-care ^1^H 3-T magnetic resonance equipment.
- Normalised R2 relative dynamic changes after the intravenous injection of a H_2_^17^O saline solution provide a unique opportunity to map water pathophysiology *in vivo*.
- The relationship between R2 and H_2_^17^O concentration was found to be linear with an initial threshold at 0.2%.
- A mean 10% change in the R2 value after the H_2_^17^O injection was shown in rats, with posterior stabilisation.

## Background

Oxygen has three stable isotopes: ^16^O, ^17^O and ^18^O, being ^16^O by far the largest component. ^17^O natural abundance is only 0.037% [1], being the only oxygen nuclei with gyromagnetic ratio (*γ* = 5.77 MHz/T) and a half-integer spin of 5/2 [2]. These properties allow magnetic resonance (MR) experiments to detect ^17^O, although the relaxation times of ^17^O are much shorter than those of the hydrogen isotope (^1^H) [3, 4]. Interestingly, ^17^O_2_ molecules are not detectable by MR neither in gas form nor dissolved in water or within oxyhaemoglobin due to their strongly paramagnetic property [5].

The direct observation of ^17^O has several limits for clinical implementation [1, 3]. Inhaled ^17^O administration and distribution have been studied with special coils adjusted to the precession frequency of ^17^O. Multinuclear MR units are restricted mainly for experimental purposes, needing specific transmission-reception coils tuned to the ^17^O resonance frequency and ultrashort echo time pulse sequences [1]. The *in vivo* ^17^O-MR images are obtained at ultrahigh fields (B_o_ of 9.4 and 7 T) [1, 3] but also at clinically used magnetic fields (B_o_ of 1.5 and 3 T) [6, 7].

^17^O can be also detected by ^1^H-MR when it is bound to protons such as in water molecules (H_2_^17^O). It is very useful because the metabolically derived H_2_^17^O could be indirectly quantified by ^1^H-MR [8]. The ^17^O consumption rate, *i.e,* cerebral metabolic rate of oxygen consumption (CMRO_2_), can be determined by the inhalation of up to 70% enriched ^17^O via MR-compatible efficient ventilator devices. These indirect CMRO_2_ measurements from low-resolution MR images with low signal-to-noise ratios have been already obtained in a small number of animal and patients studies during experimental conditions [1, 7, 9]. In real practice, oxygenation has large uncertainties as the chemical environment proportions are largely unknown, introducing biases in ^17^O detectability by ^1^H-MR [10]. The MR signal induced by ^17^O is weak compared to ^1^H due to its low gyromagnetic ratio and scarcity, which makes it difficult to quantify using direct techniques because of the poor signal-to-noise ratio and lower spatial resolution [2, 11]. The cost and complexity of these indirect studies limit its widespread use.

Fortunately, ^17^O slightly shortens the ^1^H-MR transversal relaxation times (T2) of water due to the coupling of ^1^H and ^17^O spins [1, 10]. In this way, ^17^O can be also detected by the widely used ^1^H-MR imaging T2 or T2* sequences with greater overall sensitivity [10, 12]. To further simplify the MR acquisition and open new insights, ^17^O can be incorporated within water molecules (H_2_^17^O, also known as ^17^O-labelled water), allowing imaging human pathophysiology by the analysis of water compartmentalisation. The ^17^O-labelled water visualisation can be achieved not only as the end product of the ^17^O inhalation and respiration process, as described previously, but also mainly after a H_2_^17^O bolus injection [13]. As the amount of metabolically generated H_2_^17^O during ^17^O inhalation is small and variable [3], indirect ^1^H-MR ^17^O-labelled water imaging remains the best option in clinical practice. Indirect H_2_^17^O MR imaging might allow to visualise the dynamics of administered water distribution within tissues.

H_2_^17^O MR images can be obtained using T2-weighted sequences [10, 14] although proton T2 relaxation ratio maps might also afford an objective quantitative approach. This information might be crucial in many diseases, including oncology, inflammatory and degenerative diseases as aquaporins, a membrane water channels responsible for transmembrane water passage, are altered in these pathological situations [15, 16].

The aim of the study is to verify, by means of both phantom and experimental animal studies, the feasibility of indirect ^1^H-MR methods to evaluate the effect of H_2_^17^O on the MR relaxation rates by using conventional 3 Tesla MR imaging equipment and voxel-wise relaxation rates.

## Methods

First, different MR pulse sequences able to measure tissue relaxation times in standard-of-care MR clinical scanners were evaluated to select the one able to depict ^1^H-MR relaxation rate changes related to the presence of H_2_^17^O in vials with different concentration samples. Then, a pilot experimental animal study was performed to *in vivo* evaluate the tissue dynamic changes related to the intravenous administration of the labelled water in rats.

### MR imaging

^1^H-MR exams were performed on a 3-T Philips Achieva TX standard-of-care clinical system (Philips Healthcare, Best, The Netherlands) within our experimental research platform. An eight-channel array receive surface coil was used for the phantom studies and an eight-channel volume wrist coil was employed for the rat studies.

The T1, T2*, and T2 relaxation times and respective relaxation rates were calculated from the H_2_^17^O phantoms. Details of the T1, T2*, and T2 MR sequences are summarised in Table 1.

**Table 1:** Magnetic resonance sequences used for the relaxation times calculations

Sequences	Echo time (TE); repetition time (TR)	Flip angle	Acquisition matrix; voxel size (mm)	SENSE acceleration factor; number of acquisitions
Three-dimensional T1-weighted gradient-echo with variable flip angle	TE = 4.6 ms; TR = 14 ms	5 different (5°, 10°, 15°, 20° and 45°)	192 × 192 × 15; 1.88 × 1.88 × 5	2; 1
Two-dimensional T2*-weighted gradient-echo	TR = 13; 12 TEs. Two different acquisitions with different echo time intervals (1, 2, 3, 4, 5, 6, 7, 8, 9, 10, 11, 12 ms; and 15, 17.8, 20.6, 23.4, 26.2, 29, 31.8, 34.6, 37.4, 40.2, 43, 45.8 ms)	10°	96 × 96 × 13; 1.88 × 1.88 × 5	1.8; 1
Two-dimensional T2-weighted turbo spin-echo	TR = 800; 8 TEs. Four different acquisitions with different echo time intervals (10, 20, 30, 40, 50, 60, 70, 80 ms; 20, 40, 60, 80, 100, 120, 140, 160 ms; 40, 80, 120, 160, 200, 240, 280, 320 ms; 50, 100, 150, 200, 250, 300, 350, 400 ms)	90°	96 × 96 × 1; 1.67 × 1.67 × 5	1; 1

*SENSE* Sensitivity encoding parallel imaging method

### Phantom study

**Fig. 1:**
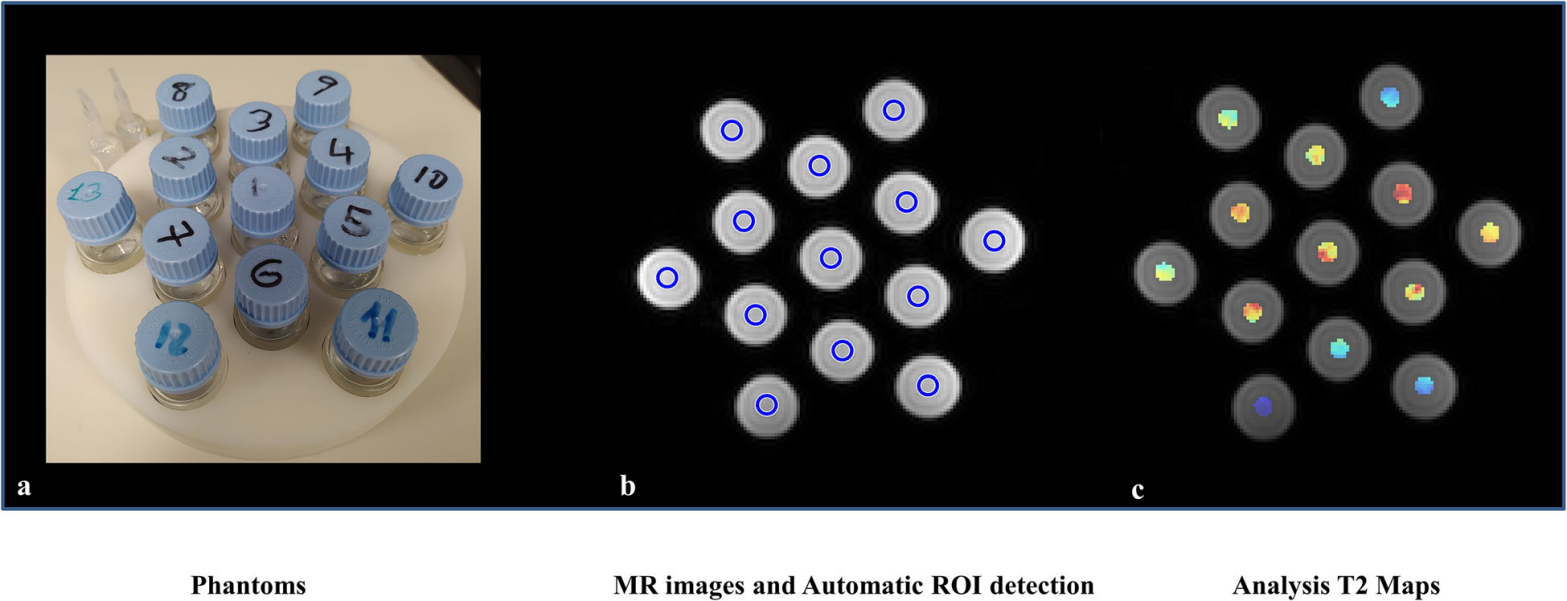
Phantom (**a**), magnetic resonance images (**b**), and T2 parametric maps (**c**) obtained in the study with the automated regions-of-interest selection within the tubes

The in-house phantom consisted of a cylinder containing 13 internal holes into which 50 mL vials could be inserted. Different concentrations of enriched H_2_^17^O water were used to prepare the saline solution by adding sodium chloride to 0.9% (Fig. 1). Nineteen vials were prepared with increasing H_2_^17^O concentrations: 0.037 (natural abundance), 0.043, 0.045, 0.050, 0.074, 0.093, 0.145, 0.245, 0.491, 0.881, 1.270, 1.603, 2.0, 2.5, 3.1, 3.8, 4.6, 5.5, and 6.5%. Because there were more vials than holes in the cylinder, two phantoms were used, each one with a different set of H_2_^17^O concentrations.

All prepared phantoms with the different set of H_2_^17^O concentrations had three tubes of standard saline fluid as control, representing the 0.037% natural abundance of H_2_^17^O (20.56 μmol/g of water) [3, 10]. The two phantoms were exam 30 times with the three MR sequences for the R1, R2*, and R2 calculations. The location of the phantom was slightly different in each acquisition, in order to average the inhomogeneity of RF and coil sensitivity in the acquired images.

### Animal study

Eight female Wistar rats (mean weight, 292 ± 32 g, mean ± standard deviation) were initially anaesthetised with isoflurane (5%) using an induction box. During the MR acquisition, rats kept anaesthetised with sevoflurane (3%) through a face mask.

The rats were positioned on prone position inside the wrist coil. The multi-echo turbo spin-echo (TSE) sequence with 20-ms echo time intervals was selected according to the phantom results (higher changes and rho Spearman correlation coefficient) with the following 8 echo times: 20, 40, 60, 80, 100, 120, 140, and 160 ms.

A venous tail cannula was used to slowly inject H_2_^17^O to the rats after the baseline MR acquisition. The volumes of the 70% H_2_^17^O saline solution were selected according to the weight of each rat to achieve an intravascular dose of 4.6%, assuming a rat blood volume of 65 mL/kg and according to the phantom results and similar previous studies [13]. The H_2_^17^O concentration injected to the rats was the one with the highest statistical differences with the saline solution (0.037%). The injection duration lasted 30 s plus a small bolus of 1 mL of standard saline, which also lasted 30 s.

The concatenated dynamic multiecho MR acquisitions consisted of a baseline acquisition and 82 consecutive series immediately after the slow H_2_^17^O injection. Each dynamic series lasted 3 min and the overall sequence duration was 246 min.

The experimental protocols were approved by the Institutional Animal Ethics Committee of our Research Institute and performed in accordance with our national and institutional regulations.

### Image analysis

MR images from phantoms and rats were acquired in Digital Imaging and Communications in Medicine, DICOM, format and converted into Neuroimaging Informatics Technology Initiative, NIFTI, format files. All images were processed and analysed using an in-house software application developed in MATLAB release 2018b (Mathworks Inc., Natick, MA, USA) to perform the quantitative R1, R2*, and R2 analysis of each acquisition.

Relaxation rates calculation from the *in vitro* phantom experiments were performed to demonstrate the influence of different H_2_^17^O concentrations on the different magnetic relaxation rates (R1, R2*, and R2). The phantom processing pipeline included an automatic detection of a circular region of interest within the vials, with an area of 118 mm^2^, located at the centred 5 slices (Fig. 1). The T1-weighted variable flip angles images were fitted to the signal intensity equation (Equation 1) for GRE sequences to extract the longitudinal relaxation T1 and derived R1 (1/T1) map [17]. The GRE equation function was used to fit and extract T1 values.

1 $$ {M}_z\left({\uptheta}_n\right)={M}_0\frac{1-{e}^{-\frac{TR}{T_1}}}{1-\cos \left({\uptheta}_n\right){e}^{-\frac{TR}{T_1}}}\sin \left({\uptheta}_n\right) $$

M_0_, magnetisation in balance when the net magnetisation vector points in the direction of the applied magnetic field B_o_; T1, time elapsed until the difference between longitudinal magnetisation (M_z_) and its equilibrium value (M_0_) is reduced by an e factor; TR, repetition time; θ_n_, tilt angle.

On the other hand, an exponential model [18] was adjusted to pixel intensities echo by echo by curve fitting to generate the T2* and T2 maps (Fig. 1). All fitting processes were obtained using the Levenberg-Marquardt algorithm. The GRE and spin echo equation functions (Equation 2) were used to fit and extract T2* and T2 values, SI being the voxel signal intensity. For the accurate quantification of T2, all echoes were used.

2 $$ SI=k{e}^{-\frac{TE}{T{2}^{\ast }}}\kern1em \mathrm{and}\kern1em SI=k{e}^{-\frac{TE}{T2}} $$

The R1, R2*, and R2 ratio values were then obtained (1/relaxation time) by pixel wise averaging.

The *in vivo* image analysis of the rats’ brains was calculated only for the R2 relaxation rate, as this was the one with a relationship with H_2_^17^O presence and quantity. The processing pipeline for all the rats included a manual segmentation of the brain at the central slice of the multi-echo T2-weighted sequence. The T2 relaxation time values were obtained voxel by voxel and the R2 (1/T2) parametric maps were calculated. Also, the relative R2 decay, expressed as the ratio between the R2 value before (PRE) and after (POST) contrast administration and normalised to the R2 PRE value, were obtained for all the time points after the contrast administration following the expression: (R2POST minus R2PRE)/R2PRE. Normalised ratios were also used to calculate the parametric maps for the first time point, obtained 3 min after the end of the intravenous labelled water administration, as this was the one with a maximum change in the relaxation ratio.

### Statistical analysis

The statistical comparisons for signal change, ^17^O-labelled water concentration and MR sequences were performed with SPSS®, version 24.0 (IBM, NY, USA). Results were obtained as the median and interquartile range (IQR) of the ratios all over the concentration values and for the different relaxation ratios. The normality of the distributions was evaluated with the Shapiro-Wilk test. The Spearman rho correlation coefficients between *in vitro* concentrations and relaxation ratios were also calculated. A Games-Howell post hoc test to detect statistical differences for the relaxation ratios values between concentrations was used. A *p* value lower than 0.05 was considered indicative of a statistically significance.

## Results

### MR sequence selection for the indirect H_2_^17^O imaging and quantitation

The influence of the different H_2_^17^O concentrations on R1, R2 and R2* values were evaluated 30 times with all the H_2_^17^O steps (from 0.037% to 6.5%). The distributions were not normal for the R1, R2*, and R2 experiments (Shapiro-Wilk test, *p* < 0.001).

There were no statistically significant changes on the R1 values with increasing H_2_^17^O concentrations between any concentration (0.48 s^−1^, IQR 0.24 s^−1^). The Spearman correlation was low (rho = 0.148) with no significant changes on R1 with the different-labelled water concentrations (*p* = 0.075, Games-Howell post hoc test). Similar results were obtained for the R2* experiments and the two sequences having different echo time intervals. R2* values (17.31 s^−1^, IQR 27.87 s^−1^) showed a low Spearman correlation coefficient (rho = 0.36) and no statistically significant changes between the different concentrations were observed (*p* = 0.739).

However, the R2 relaxation rate values showed a statistically significant linear increase with increasing H_2_^17^O concentration. The R2 value obtained with the TSE sequence using 40 ms echo interval showed a significant change (*p* < 0.001) with concentration (0.57 s^−1^, IQR 0.133 s^−1^) and an increased in the Spearman correlation coefficient (rho = 0.539). The R2 having the higher statistical difference (0.67 s^−1^, IQR 0.34 s^−1^, *p* < 0.001) and Spearman correlation (rho = 0.79) was obtained with the TSE T2-weighted sequence with 20-ms echo time interval. The first 9 lowest concentrations did not show a statistical difference with the natural abundance 0.037% H_2_^17^O concentration. The highest significant R2 differences were obtained for the higher concentrations (3.1–5.5%). The R2 reached the highest value at the higher 5.5% concentration. The R2 increase was adjusted to a linear fit between 0.25% and 5.5%, represented with the following equation:

$$ \mathrm{R}2=0.405\cdotp \mathrm{Concentration}+0.3215 $$

### H_2_^17^O concentration selection

**Fig. 2:**
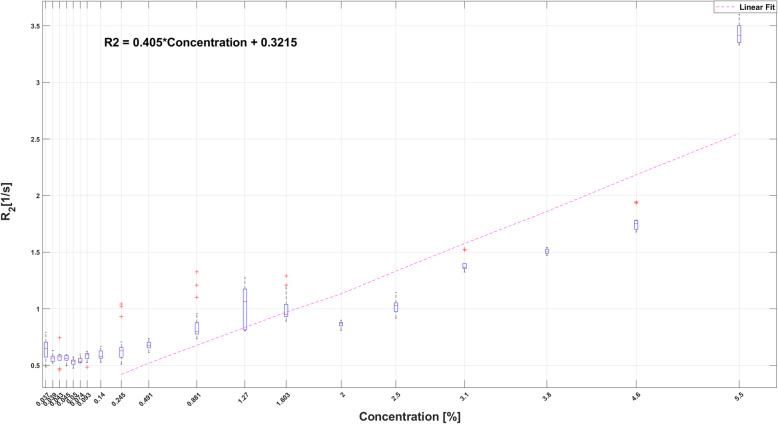
Boxplot of the R2 relaxation rates and H_2_^17^O concentrations as measured with the phantom studies. The linear fitting and equation are shown

Boxplots of the R2 relaxation rates (s^−1^) obtained from the multi-echo TSE sequence with 20-ms TE interval acquisitions of the two phantoms for all the labelled water concentration are shown in Fig. 2. The R2 relaxation ratios showed a linear signal adjustment as the H_2_^17^O concentration increases between 0.25 and 5.5%. The concentrations with a highest statistical difference (*p* values < 0.001) from the saline solution were from 3.1 to 5.5%. In this sense, the pairwise comparisons also showed the highest significant difference (*p* values < 0.001) between the five higher concentrations (3.1–5.5%). Any of these five concentrations could therefore be used in the rats’ experiments to measure the induced relaxation time R2 changes of the tissues.

### Animal studies

For the animal studies, the multi-echo TSE sequence with 20-ms TE interval was selected for its highest signal. The H_2_^17^O selected dose was chosen according with the weight of the rat to have a final intravascular solution of 4.6% (from an expected 20 mL of blood per rat). This dose and concentration were obtained by using the 70% enriched H_2_^17^O vial. This value was close to the highest one in the phantom studies and was selected to guarantee to observation of signal changes. No animal showed any clinically evident adverse effect related to the 70% enriched H_2_^17^O administration.

**Fig. 3:**
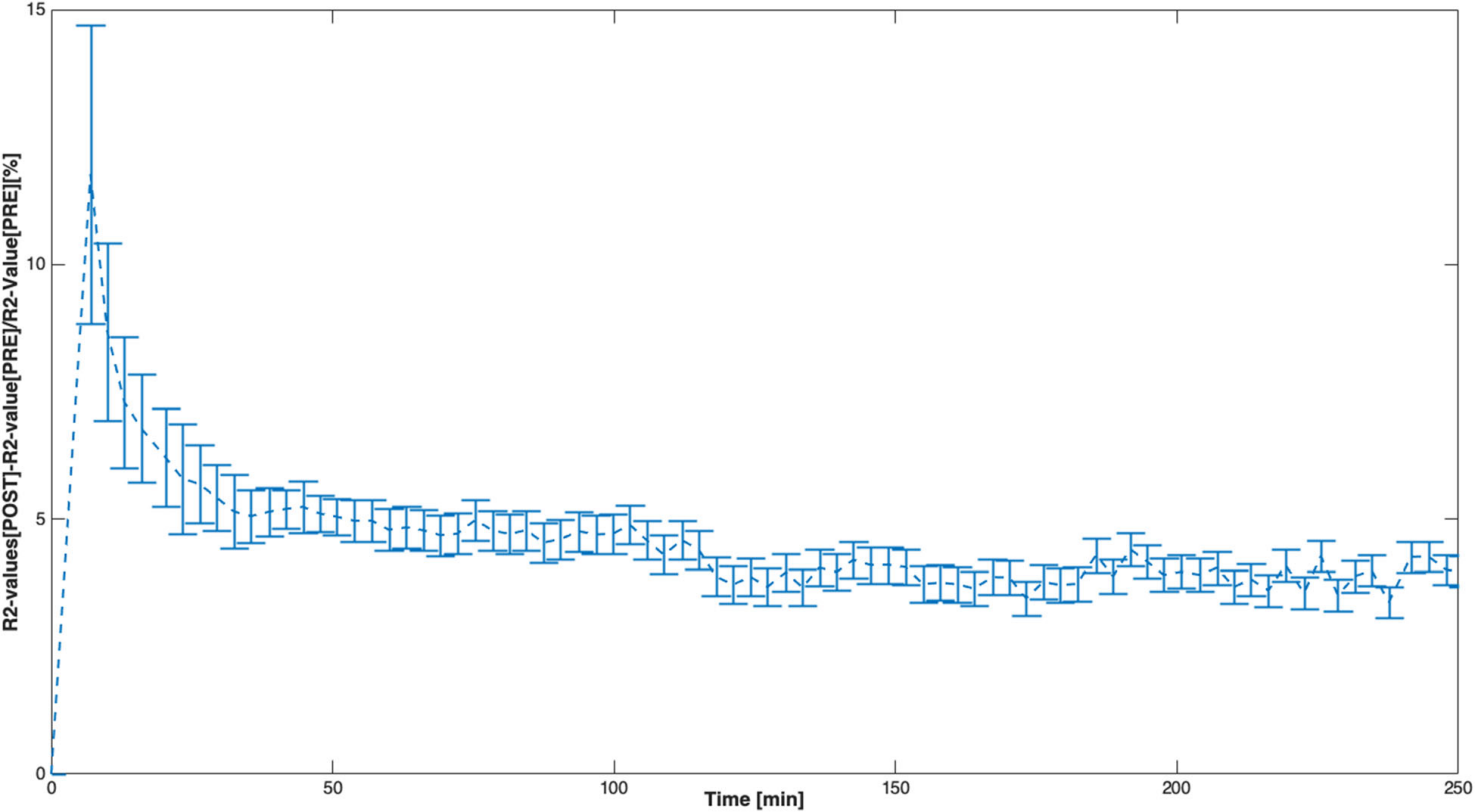
Mean relative normalised R2 values of the rat brain over the 246 min. Time 0 corresponds to the precontrast magnetic resonance images

**Fig. 4:**
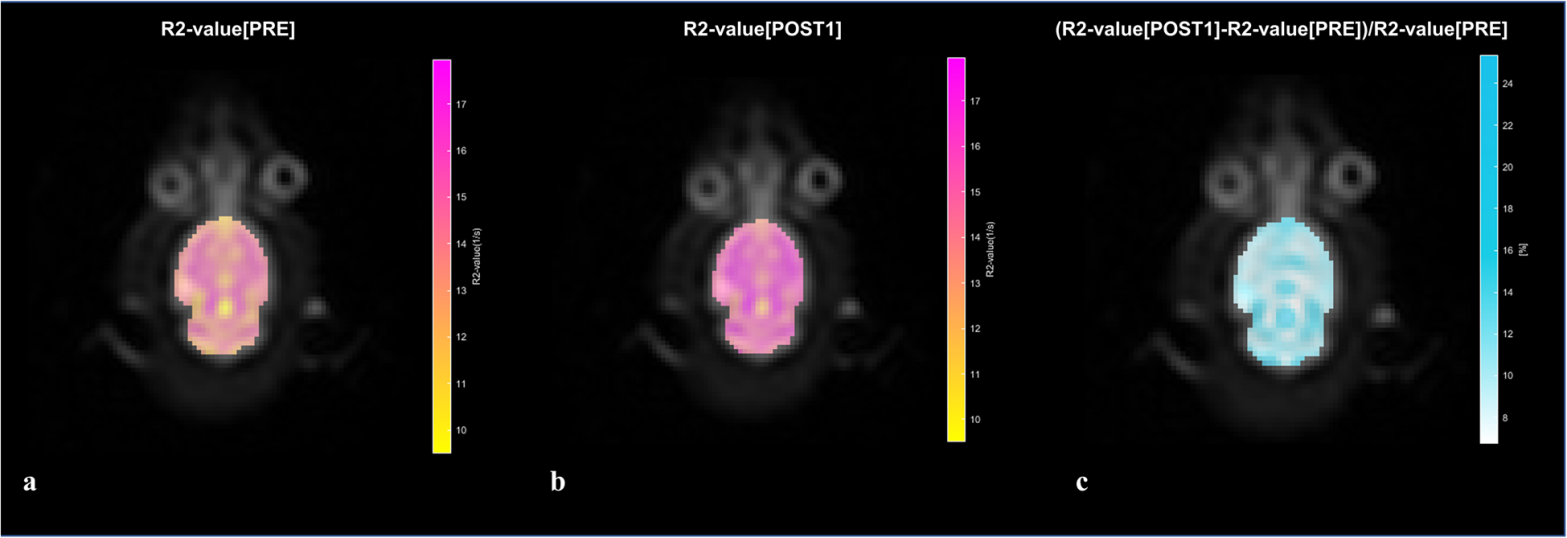
R2 parametric maps of the rat brain before (PRE), after (3 min, POST1) and relative normalised change (R2POST1 minus R2PRE)/R2PRE). The normalised R2 changes reflect the local distribution of labelled water

In Fig. 3, the evolution curves of R2 value normalised to the baseline R2 value for each rat is shown. This curve shows a mean 10% change in the R2 value immediately after the H_2_^17^O was injected into the rat to a gradual decrease towards baseline R2 values. Figure 4 shows R2 maps of a rat’s brain at baseline and after injection of H_2_^17^O. In addition, it also shows the R2 values normalised to the baseline.

## Discussion

Although not in clinical use, ^17^O is a natural magnetic isotope that allows enriched labelled water to be traced by standard of care MR imaging. Our study shows that an indirect ^1^H-MR imaging method is able to measure H_2_^17^O-related changes by using R2 ratios from conventional MR equipment and sequences.

Our phantom study demonstrates that an increased concentration of H_2_^17^O results in an increase in the R2 relaxation rate, which was also confirmed in the experimental animal study. These results endorse the indirect correlation between the H_2_^17^O concentration and R2 values [3] and the absence of R1 modifications [14, 19, 20]. The non-significant correlation found with R2* values was not published before. Although R2 linear relationships were obtained in previous studies [19], the relationship found in this study used a higher concentration range and showed a linear behaviour between 0.25 and 5.5%. This 0.25% threshold was not reported before [19].

Most research groups have used mainly T2-weighted steady-state free precession and echo-planar sequences, with or without radiofrequency irradiation to remove the residual ^1^H-^17^O scalar coupling [20, 21]. However, our multi-echo TSE T2-weighted sequences allow to obtain high spatial resolution R2 relaxation rate parametric maps and normalised R2 variation maps, probably improving more objective correlations. Other experiments using T1ρ weighted images for dynamic ^1^H-MR analysis of perfusion have also been published, but not replicated [22, 23].

The R2 relaxation rate measurement allows to observe the correlation to labelled water concentrations. The relationship between the different H_2_^17^O concentrations and R2 values allows a depiction of injected water in the range between 0.25 and 5.5% by the change in the relaxation rate. The dependence of the H_2_^17^O concentration and R2 relaxation rate has also been experimentally proven in biological solutions up to 5% enrichment [3]. Our results using the R2 relaxation rate can be more consistent than previously published methods using balanced steady state T2/T1 and T1ρ-weighted sequences. Although balanced steady-state free precession and echo-planar imaging sequences have been used in MR animal and human experiments, our R2 calculation approach by using a multi-echo TSE sequence seems to be sensitive to small changes, being able to discriminate between a range (0.25–5.5%) of concentrations at a cost of a lower temporal resolution (3 min per dynamic series) [20]. The signal change and spatial resolution of R2 maps allow to evaluate water kinetics in different organs and structures with 3-T magnets [6, 12, 14].

Assuming that humans have a 60% total body water, an intravascular concentration of H_2_^17^O around 4.5% can be obtained by injecting 2 mL/kg body weight of 70% enriched H_2_^17^O vial (38.43 molar), taking into consideration a mean total body water volume of 42 L, 3 L of intravascular water, and 11 L of interstitial water. Therefore, R2 relaxation rate parametric images might allow to depict the injected water distribution dynamics in clinical practice. To be used, the barrier to overcome is the high cost of manufacturing H_2_^17^O. Unless a cheaper process is available, close to 900€/mL of 70% enriched solution clearly limits its clinical use (data provided by NUKEM Isotopes GmbH, Germany). As an alternative, oxygen transfer by peroxides (H_2_O_2_) has been proposed as a low-cost method for ^17^O synthesis, amongst others [24]. Relaxometry protocols are more sensitive so smaller changes in R2 will also allow to measure lower H_2_^17^O concentrations.

Patients’ safety must be considered with this contrast agent. ^17^O is a weak proton-relaxing agent that must be used at relatively high concentrations (4–5%). As expected, there is no publication reflecting that H_2_^17^O is different in toxicity than ordinary water, being a natural constituent of all living systems.

The relative R2 variations can be converted into H_2_^17^O concentration changes in a voxel-by-voxel approach (see Fig. 4). The R2 dynamic acquisition would allow the pharmacokinetic modelling analysis of the signal behaviour to assess the movement of labelled water within the different compartments after H_2_^17^O-enriched physiological saline solution administration [14]. Molecules that integrate living organisms, such as ions, sugars or proteins are dissolved in an aqueous medium. Cells exchange molecules through their membranes using mechanisms such as passive diffusion or specific transport and channel proteins. The movement of water into and out of cells is a fundamental biological process that is essential for life. The water molecule is neutral, water movement across the cell membrane being not just by simple diffusion but mainly by water channels. These proteins were originally named CHIP28 (“channel-forming integral protein”), but they are now known as aquaporins (AQP) [15, 16]. These membrane water channels have currently 13 different forms in mammals. AQP have a critical role in preserving cell stability and integrity all over the body [16, 25]. Functional *in vivo* studies of the AQP using a non-invasive technique, such as H_2_^17^O labelled MR imaging and R2 parametric maps, could offer a unique relevant opportunity for the depiction of AQP changes resolved in space (R2 variation parametric maps) and time (dynamic and follow-up studies) [14]. Although AQP can be histologically evaluated, *in vivo* AQP dynamic kinetics modifications induced by disease, including degeneration and cancer proliferation, cannot be evaluated without the use of a water tracer [15, 16]. It seems therefore reasonable to further improve the R2 maps methodology avoiding possible biases, providing images with even smaller voxel size, and sampling the water dynamic changes faster. Expected areas of specific interest for this analysis are related to a wide variety of diseases, including cancer, renal dysfunction, neurological disorder, epilepsy, metabolic syndrome, infection, and cardiac diseases [25].

A possible bias in the quantitation of H_2_^17^O with the indirect method is that both ^1^H-^17^O scalar coupling and chemical exchange between H_2_^17^O and H_2_^16^O are sensitive to pH and temperature [3]. However, the use of a normalised R2 change ratio minimised this bias. Also, as the rats in the *in vivo* experiment might change their temperature by a few degrees, and this might slightly influence R2, we do believe that this variation will have a minimal impact on the calculated relaxation rates. One main advantage of intravenous administrated H_2_^17^O over the inhalation of ^17^O_2_ is that the amount of metabolically generated H_2_^17^O from ^17^O_2_ inhalation is unknown and usually less than the amount of administrated H_2_^17^O [3]. Although field strength can be a limiting factor, fortunately the use of indirect methods requires lower magnetics fields than direct methods to the quantification H_2_^17^O [3]. Also, the achieved spatial resolution can be considered sufficient (1.67 × 1.67 mm in plane) (see Fig. 4), but the temporal resolution was limited due to the long TR and TE multi-echo sequence. A much faster resolution might be required for a detailed examination of water kinetics in small structures. Maybe the use of accelerated artificial intelligence driven sequences will help to avoid this limitation.

This study had some other limitations that should be considered. First, the number of rats in this work is reduced. Second, our study only evaluated relaxation rates using variable flip angles sequences, GRE and TSE for measure T1, T2*, and T2, respectively. Other alternative methods, such as saturation/inversion recovery sequences with variable TI, should be investigated. Unfortunately, estimating the H_2_^17^O concentrations from the change in R2 values is quite challenging. Although a change in R2 value can be extrapolated to a H_2_^17^O concentration, we did prefer to normalise this change to the precontrast R2 to standardise measurements. These normalised results will allow the validation with other MR equipment and vendors. It will be relevant to evaluate the best approach (normalised ratios *versus* concentrations) regarding reproducibility and explainability of results.

In summary, we have provided proof of concept that ^1^H-MR images allow to detect ^17^O-enriched H_2_O molecules by the induced changes in R2 of water protons at standard-of-care MR scanners without dedicated hardware. Detection of intravenous administered H_2_^17^O dynamics could potentially provide new insights into water distribution. Normalised R2 dynamic changes after the intravenous injection of H_2_^17^O saline solution add important insights into the *in vivo* evaluation of water kinetics modifications by disease at clinically available 3-T MR scanners.

## Data Availability

The data generated and/or analysed during the current study are not publicly available due institutional limitations but are available from the corresponding author on reasonable request.

## Abbreviations

AQP: Aquaporins
CMRO_2_: Cerebral metabolic rate of oxygen
GRE: Gradient-echo
IQR: Interquartile range
MR: Magnetic resonance
R1: Relaxation rate T1
R2*: Relaxation rate T2*
R2: Relaxation rate T2
TE: Echo time
TR: Repetition time
TSE: Turbo spin-echo
